# A PET/CT nomogram incorporating SUVmax and CT radiomics for preoperative nodal staging in non-small cell lung cancer

**DOI:** 10.1007/s00330-020-07624-9

**Published:** 2021-02-09

**Authors:** Yunming Xie, Hongguang Zhao, Yan Guo, Fanyang Meng, Xiangchun Liu, Yiying Zhang, Xiaochen Huai, Qianting Wong, Yu Fu, Huimao Zhang

**Affiliations:** 1 Department of Radiology, The First Hospital of Jilin University, No.71 Xinmin Street, Changchun, 130012 Jilin China; 2 Department of Nuclear Medicine, The First Hospital of Jilin University, Changchun, Jilin China; 3 GE Healthcare, Shanghai, China; 4 Philips Healthcare, Beijing, China

**Keywords:** PET-CT, Nomograms, Lymphatic metastasis, Non-small cell lung cancer

## Abstract

**Objectives:**

To develop and validate a PET/CT nomogram for preoperative estimation of lymph node (LN) staging in patients with non-small cell lung cancer (NSCLC).

**Methods:**

A total of 263 pathologically confirmed LNs from 124 patients with NCSLC were retrospectively analyzed. Positron-emission tomography/computed tomography (PET/CT) examination was performed before treatment according to the clinical schedule. In the training cohort (*N* = 185), malignancy-related features, such as SUVmax, short-axis diameter (SAD), and CT radiomics features, were extracted from the regions of LN based on the PET/CT scan. The Minimum-Redundancy Maximum-Relevance (mRMR) algorithm and the Least Absolute Shrinkage and Selection Operator (LASSO) regression model were used for feature selection and radiomics score building. The radiomics score (Rad-Score) and SUVmax were incorporated in a PET/CT nomogram using the multivariable logistic regression analysis. The performance of the proposed model was evaluated with discrimination, calibration, and clinical application in an independent testing cohort (*N* = 78).

**Results:**

The radiomics scores consisting of 14 selected features were significantly associated with LN status for both training cohort with AUC of 0.849 (95% confidence interval (CI), 0.796–0.903) and testing cohort with AUC of 0.828 (95% CI, 0.782–0.919). The PET/CT nomogram incorporating radiomics score and SUVmax showed moderate improvement of the efficiency with AUC of 0.881 (95% CI, 0.834–0.928) in the training cohort and AUC of 0.872 (95% CI, 0.797–0.946) in the testing cohort. The decision curve analysis indicated that the PET/CT nomogram was clinically useful.

**Conclusion:**

The PET/CT nomogram, which incorporates Rad-Score and SUVmax, can improve the diagnostic performance of LN metastasis.

**Key Points:**

*• The PET/CT nomogram (Int-Score) based on lymph node (LN) PET/CT images can reliably predict LN status in NSCLC.*

*• Int-Score is a relatively objective diagnostic method, which can play an auxiliary role in the process of clinicians making treatment decisions.*

**Supplementary Information:**

The online version contains supplementary material available at 10.1007/s00330-020-07624-9.

## Introduction

Lung cancer is considered the leading cause of cancer death worldwide. In patients with non-small cell lung cancer (NSCLC), nodal staging (N staging) is usually closely associated with the prediction of prognosis [1–4] and clinical therapeutic planning [5, 6]. Previous studies [7–10] have found that N staging after induction therapy for stage IIIA lung cancer may determine patient survival. For advanced patients with positive N2, preoperative downstaging from N2 to N0 can improve the 5-year survival rate for up to 35.8%, while those with a persistent tumor in their lymph nodes (LNs) (N1 and N2) tend to have a 5-year survival of 9% [8]. Therefore, accurate preoperative clinical N staging and restaging is the key to clinically individualized decision making, which may enable patients to receive more effective treatment and improve prognosis.

^18^F-Fluorodeoxyglucose positron-emission tomography/computed tomography (^18^F-FDG PET/CT) is considered to be the most reliable functional imaging method for assessing the status of mediastinal and hilar LNs [11, 12]. This non-invasive method can simultaneously provide metabolic and anatomical information. Metastatic LN is usually accompanied by tumor cells’ active metabolism of ^18^F-FDG and lymphadenopathy, increased standard uptake value (SUV) [13]. It often appears as a bright light spot on PET images and enlarged in size as round lesions on CT images, mainly in short-axis diameter (SAD) [14]. ^18^F-FDG PET has become a standard imaging procedure for N and M staging in NSCLC. However, due to the complications, such as inflammatory, granulomatous, and infectious diseases, highly sensitive PET may detect false-positive LNs [15–18]. Low spatial and organizational resolution of PET images, the small size of LNs, and inevitably cardiac and breathing motion artifacts during the long duration of the PET imaging process result in a missed diagnosis of small-sized metastatic LNs. Moreover, CT is the most widely used clinical examination. SAD ≥ 1 cm is often used as the diagnostic criterion. However, normal, inflammatory proliferative, and metastatic LNs partly overlap in size, and normal LNs vary in size and shape [19, 20], which results in a missed diagnosis of micro-metastatic LNs and misdiagnosis of inflammatory hyperplastic LNs, leading to an increase in the false-negative rate. Therefore, there is an urgent need to develop a more effective method to predict LN status preoperatively.

Radiomics is a medical image (PET, CT, and MRI) method based on high-throughput extraction of quantitative image features, which is used to predict underlying tumor biology and behavior [21, 22]. Over the last decade, a variety of prediction models [23–26] have been established using primary tumor as the region of interest (ROI) based on the changes in the tumor-induced microenvironment [27–29], which can be used to classify LN metastasis in N^−^ or N^+^, thus providing a qualitative diagnosis of LN metastasis of cancer, including lung cancer, colorectal cancer, breast cancer, and others fields. However, with the promotion of precision medicine, qualitative diagnosis of LN metastasis still does not meet the requirement for individualized treatment; thus, quantitative N staging has become of urgent importance in clinical practice.

To the best of our knowledge, so far, there is a rare correlated study that investigated high-latitude radiomics features and maximum standard uptake value (SUVmax) based on LNs for prediction of N staging in NSCLC. Therefore, the objective of our study was to establish and validate a PET/CT nomogram that incorporates the metabolic information (SUVmax) and structural information (radiomics features) of LNs for preoperative quantitative estimation of LN metastasis that could further help clinicians in making individualized therapeutic decisions and predict prognosis.

## Materials and methods

### Study design

**Fig. 1:**
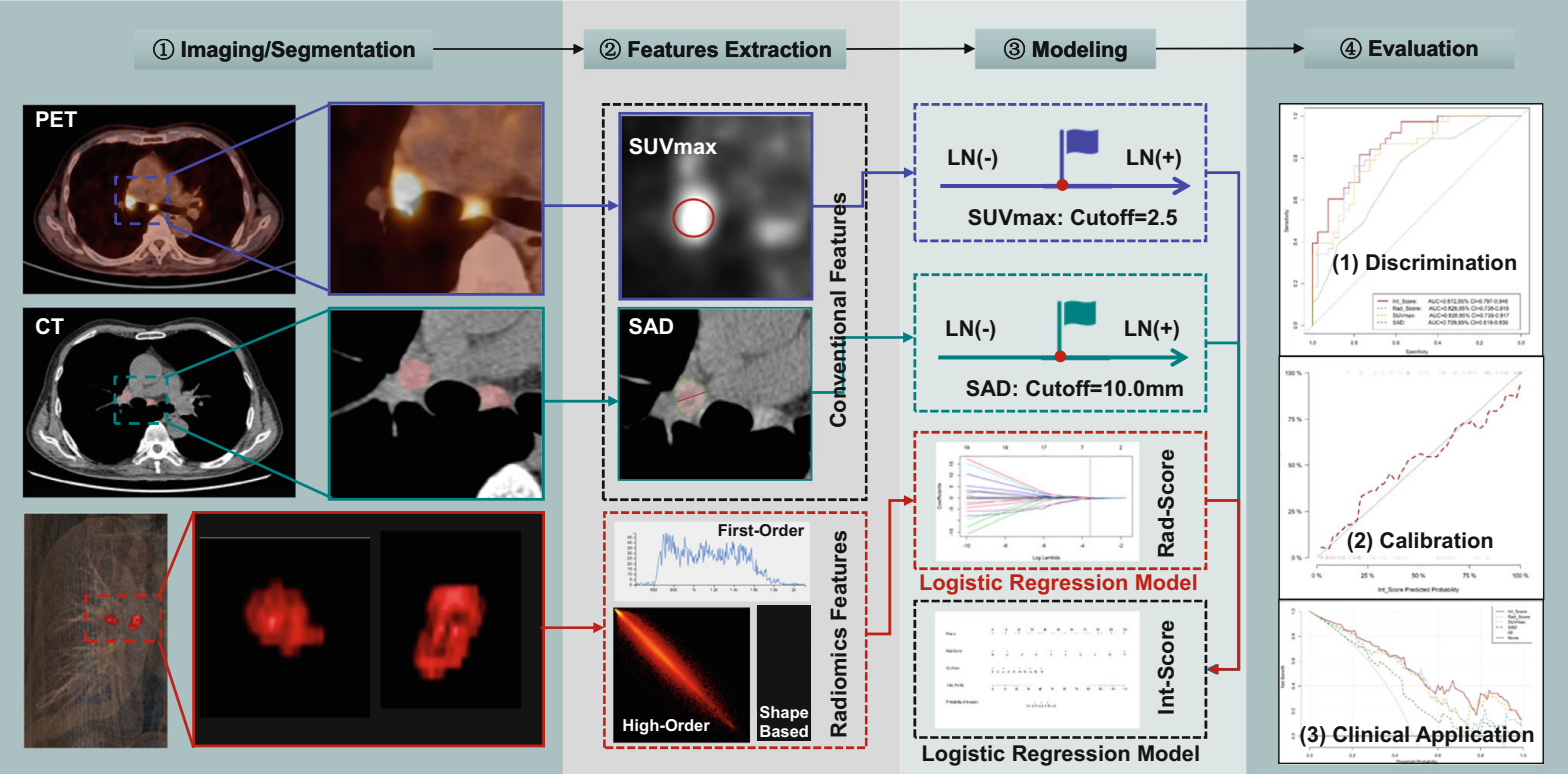
Workflow diagram of the study. In the first row, the blue solid border figures represent the SUVmax of LNs measured on PET image; the blue dashed border plot represents SUVmax single factor prediction model established with a cutoff value of 2.5. In the second row, the green solid border figures represent the short-axis diameter (SAD) of LNs measured on CT image; the green dashed border plot represents the SAD single-factor prediction model established with a cutoff value 10.00 mm. In the third row, the red solid border figures represent that VOIs segmented based on LNs; the red dashed border plot at the bottom the second column represents the radiomic features of LNs including first-order, shape, and high-order features, and the red dashed border plot in the third column represents the radiomics model (Rad-Score) built by LN status-related radiomics features using multivariable logistic regression analysis. The black dashed border plot at the bottom of the third column represents the PET/CT nomogram (Int-Score) incorporated SUVmax and Rad-Score by multi-variable logistic regression analysis. In the last column, these three plots represent the receiver operating characteristic (ROC) curves, calibration curves, and decision curve analysis (DCA) curves in the testing cohort in order from top to bottom

The institutional review board has approved this single-center study. A comprehensive workflow diagram of this study is presented in Fig. 1.

### Patients

A total of 124 NSCLC patients (male:female = 60:64; average age, 58.5 ± 0.73 years; range, 33–74 years) were retrospectively enrolled in this study between January 2017 and May 2019. The inclusion criteria were as follows: (1) NCLSC pathologically confirmed by surgery or biopsy; (2) lymphadenectomy or lymph node biopsies were performed, and the pathological reports were obtained; (3) preoperative ^18^F-FDG PET/CT scans were performed (the interval between PET/CT scans and surgery or biopsy was less than 2 weeks). The exclusion criteria were as follows: (1) preoperative history of other malignancies besides lung cancer; (2) poor image quality.

**Fig. 2:**
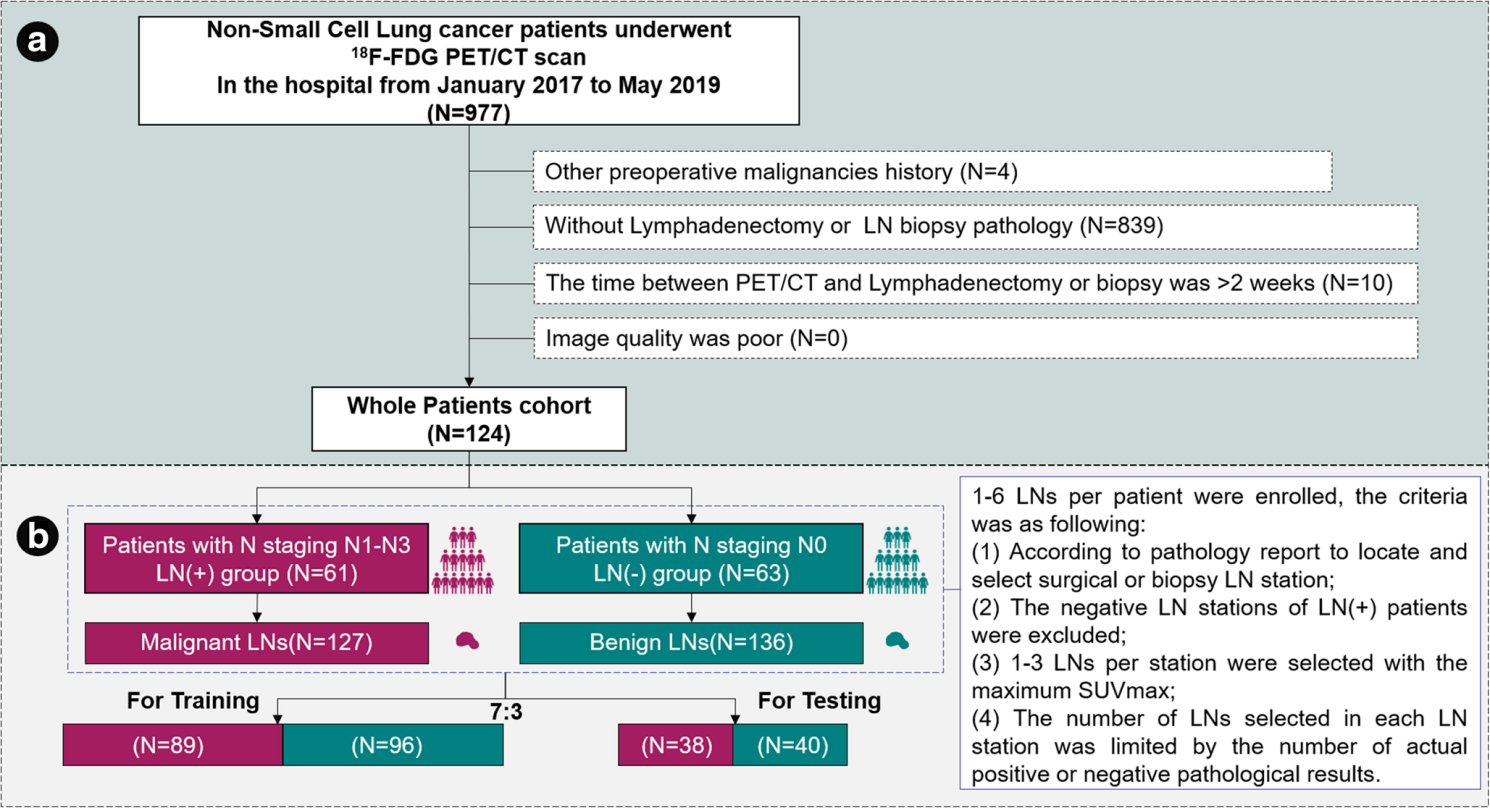
Flow diagram of the selection of patients (**a**) and LNs (**b**)

The patients who satisfied the inclusion criteria were identified for the whole patient cohort, as shown in Fig. 2a. A total of 110 patients underwent surgical resection; LN pathology results from supra-clavicular LN biopsy pathway were obtained from 14 patients. There were 93 (75.0%) patients with adenocarcinoma, followed by squamous cell carcinoma (*n* = 29, 23.4%), and few other histological subtypes of lung cancer (*n* = 2, 1.6%). There were 63 patients with N0 stage, 27 patients with N1 stage, 20 patients with N2 stage, and 14 patients with N3 stage. These patients were divided into the LN(+) group and LN(−) group based on pathological reports.

The LN enrolled flowchart is shown in Fig. 2b. The patients who underwent surgery had relatively early TNM pathological stages (Supplemental Table S1). The inclusion criteria were as follows: (1) according to the pathology report defined with the N staging standard of the eighth edition [30] to locate and select surgical or supra-clavicular biopsy LN station; (2) 1–3 LNs with the maximum SUVmax were selected in the LN station; (3) the number and size of LNs selected in each LN station were limited by the number and size of actual pathological results. The exclusion criteria were as follows: the negative LN stations of LN(+) patients were excluded to ensure as much as possible the LNs selected are truly pathologically positive.

### ^18^F-FDG PET/CT acquisition and reconstruction

A Biograph 16 HR PET/CT scanner (Siemens Healthineers) was used in the single-center study. Briefly, patients were fasted for more than 6 h; and before imaging, their blood glucose levels needed to be less than 8 mmol/L. ^18^F-FDG (Sumitomo HM-12, pH 4–8, radioactive purity > 95%, radioactive concentration > 370 MBq/ml) was then intravenously injected at a dose of 3.7 MBq/kg; the image acquisition was started 1 h later. CT image scan was preformed (tube voltage 120 kV, tube current 50 mAs, rotational speed 0.5 s/r, FOV 812 mm × 812 mm, 512 × 512 matrix, slice thickness 4 mm) from the vertex to the proximal legs. Low-dose CT was used for attenuation correction, and a standard B19f soft-tissue reconstruction kernel was used for CT images. Consequently, PET image scans (8-bed positions at 2.5 min each, FOV 812 mm × 812 mm, 144 × 144 matrix, slice thickness 4 mm) were acquired from the vertex to the proximal legs with correction for dead time, scatter, and decay. PET images were iteratively reconstructed in 3D mode using ordered subset expectation maximization (2 iterations, 24 subsets, and Gaussian filtering). CT and PET images were reconstructed at a slice thickness of 2.0 mm and an increment of 1.0 mm.

### Measurement of CT radiomics features, SAD, and SUVmax

The VOIs were semi-automatically segmented using imaging post-processing software (Intellispace Discovery, Philips Healthcare) (Supplemental Appendix A-1). We segmented the LNs and measured SAD on the largest cross-sectional area of LNs. ^18^F-FDG uptake was evaluated using SUVmax.

The radiomics features were extracted using a plug-in (pyradiomics 2.1) on the IntelliSpace Discovery platform. A total of 1472 features, including shape-based features, first-order histogram features, high-order textural features, and transformed features, were obtained based on the 3D VOI (Supplemental Appendix B). The image biomarker standardization initiative (IBSI) was regarded as reference, and was taken into consideration in the radiomics features extraction and selection procedure [31].

In our department, the clinical standard for evaluating LNs on PET imaging was to classify them as PET positive when the SUVmax is ≥ 2.5, and PET negative when the SUVmax is < 2.5 [32].

### Radiomics model

With the abundance of exceptionally high-dimensional data, the Minimum-Redundancy Maximum-Relevance (mRMR) algorithm [33] and the Least Absolute Shrinkage and Selection Operator (LASSO) method [34] were used to select the most useful and strongest features in the training cohort. The principle of the mRMR algorithm is to identify the features that were highly correlated with the status of LN but had a minimum correlation with other features in order to reduce overfitting of the model. The LASSO method is suitable for the regression of high-dimensional data and can be used to select useful features with the non-zero coefficient. A radiomics score (Rad-Score) was calculated for each LN via linear combination of the selected features weighted by their respective coefficients. The performance of the built radiomics model (Rad-Score) was evaluated with discrimination, calibration, and clinical application in both training and testing cohorts.

### PET/CT nomogram

SUVmax and Rad-Score were applied to develop an integrated estimative model (PET/CT nomogram) for the status of LN in the training cohort using multivariable logistic regression analysis. Similarly, an integrated score (Int-Score) was calculated for each LN using a linear combination of the selected features weighted by their coefficients. The performance of the PET/CT nomogram (Int-Score) was evaluated with discrimination, calibration, and clinical application in both training and testing cohorts.

### Statistical analysis

All statistical analyses were performed using R Studio software (version 1.2.1335). mRMR algorithm was performed using the “mRMRe” package. LASSO regression was performed using the “glmnet” package. *p* values < 0.05 (two-sided) were considered to be statistically significant. The differences in LN-status related features between the malignant group and benign group in both training and testing cohorts were assessed by the independent *t* test or Mann-Whitney *U* test, according to the distribution type of the data. The chi-squared testing was used to compare the significance of the differences between categorical variables. The performance of the models was evaluated with discrimination, calibration, and clinical application.

#### Discrimination

Receiver operating characteristic (ROC) curves were plotted to assess the diagnostic performance of SUVmax, SAD, the Rad-Score and the Int-Score in discriminating malignant from benign LNs in training and testing cohorts. The optimal cutoff of the biomarkers calculated from the training cohort was applied in the testing cohort. The bar chart was plotted to intuitively display the discrimination performance. DeLong testing was used to compare the area under ROC curves (AUC) between training and testing cohorts.

#### Calibration

Calibration curves were plotted in both training and testing cohorts to explore the agreement between the observed outcome frequencies and predicted probabilities of the model. The Hosmer-Lemeshow testing was used to determine the goodness of fit of the models, and *p* values of more than 0.05 were considered well-calibrated.

#### Clinical application

Decision curve analysis (DCA) was used to assess the clinical usefulness of the built models by quantifying the net benefits at different threshold probabilities in the testing cohort.

## Results

### Clinical characteristics

A total of 263 LNs from 124 patients were identified in the present study and were further assigned to either the training cohort or testing cohort. Of the 263 LNs, 70% (*N* = 185) were assigned to the training cohort by stratified sampling; 89 LNs were malignant, and 96 were benign. The remaining 30% (*N* = 78) were selected for the testing cohort; 38 were malignant and 40 were benign. There was no statistically significant difference in the clinical characteristics between the LN(+) group and LN(−) group. Besides, there was no significant difference in the clinical characteristics between both cohorts, as shown in Table 1.

**Table 1:** Summary of characteristics in training and testing cohorts

Characteristics	Training cohort			Testing cohort			*p*^##^ value
	LN (+) (*N* = 89)	LN (−) (*N* = 96)	*p* value	LN (+) (*N* = 38)	LN (−) (*N* = 40)	*p* value	
Sex, no. (%)			0.075			0.581	0.910
From male patients	44 (16.73)	35 (13.31)		21 (7.98)	20 (7.60)		
From female patients	45 (17.11)	61(23.19)		17 (6.46)	20 (7.60)		
Age/mean ± SD, years	58.35 ± 6.76	59.23 ± 7.55	0.406	58.37 ± 8.22	58.55 ± 10.09	0.931	0.745
Pathology source (no. (%))			0.294			0.502	0.228
SCC	25 (9.88)	20 (6.84)		9 (3.42)	12 (4.74)		
Adenocarcinoma	62 (24.51)	76 (28.90)		28 (11.07)	28 (10.65)		
NEC	1 (0.38)	0 (0.00)		1 (0.38)	0 (0.00)		
Sarcoma	1 (0.38)	0 (0.00)		0 (0.00)	0 (0.00)		
LN station (no. (%))			0.048			0.002	0.208
#1, #2	10 (3.80%)	4 (1.52%)		7 (2.66%)	0 (0.00%)		
#3–9	32 (12.17%)	49 (18.63%)		15 (5.70%)	10 (3.80%)		
#10–14	47 (17.87%)	43 (16.35%)		16 (6.08%)	30 (11.41%)		
Lung lobe (no. (%))			0.046			0.420	0.847
Right upper lobe	25 (9.51%)	28 (10.65%)		10 (3.80%)	9 (3.42%)		
Right middle lobe	4 (1.52%)	16 (6.08%)		4 (1.52%)	5 (1.90%)		
Right lower lobe	17 (6.46%)	21 (7.98%)		8 (3.04%)	9 (3.42%)		
Left upper lobe	17 (6.46%)	14 (5.32%)		11 (4.18%)	6 (2.28%)		
Left lower lobe	26 (9.89%)	17 (6.46%)		5 (1.90%)	11 (4.18%)		
SUV_max_/median (25%, 75%)	3.95 (2.92, 6.54)	2.29 (1.51, 3.50)	*< 0.001*	4.15 (3.28, 8.68)	2.22 (3.16, 3.42)	*< 0.001*	0.759
SAD/median (25%,75%) (cm)	1.00 (0.80, 1.38)	0.90 (0.70, 1.00)	*< 0.001*	1.05 (0.90, 1.35)	0.80 (0.60, 1.08)	*< 0.001*	0.754
Rad-Score	0.582 (− 0.221, 1.892)	− 0.808 (− 1.779, − 2.000)	*< 0.001*	0.702 (0.255, 1.991)	− 0.677(− 1.230, 0.228)	*< 0.001*	0.242
Int-Score	1.387 (− 0.014, 3.109)	− 1.438 (− 2.402, − *0*.293)	*< 0.001*	1.405 (− 0.030, 3.060)	− 1.297 (− 2.422, − 0.119)	*< 0.001*	0.449

The values in italics indicate the cut-off values used in the diagnosis process

*p* < 0.001 indicates statistical significance

*SCC*, squamous cell carcinoma; *NEC*, neuroendocrine carcinoma

Lymph nodes and tumors were located on the same side of the trachea or esophagus

Lung lobe represents the tumor location

*p*^##^ value indicates the significance of differences between the characteristics in training and testing cohorts

*p* value indicates the significance of differences between the LN (+) group and LN (−) group

### SUVmax and SAD

The SUVmax (*p* < 0.001) and SAD (*p* < 0.001) were statistically different between the LN(+) group and LN(−) group. There was no significant difference between both cohorts (Table 1)**.**

### Int-Score

A multivariate logistic regression analysis was conducted to integrate the Rad-Score (Supplemental Appendix C), SUVmax, and SAD. There was a significant difference in Rad-Score, SUVmax, and SAD between LN(+) and LN(−) groups (*p* ˂ 0.001; Table 1). Considering the SAD redundancy, only SUVmax and Rad-Score were used (named Int-Score). Calculation of the Int-Score was performed using the formula: Int-Score = 5.40 × Rad-Score + 3.64 × SUVmax + (−3.31). To ensure that the model was easy to use, we presented it as a nomogram (Fig. S4, Appendix A-2). Int-Score was significantly higher in LN(+) group (Table 1**)**.

### Performance of the model

#### Discrimination

ROC curves of SUVmax, SAD, Rad-Score, and Int-Score were plotted to assess the diagnostic performance, as shown in Fig. S5 (Appendix A-2). The diagnostic efficiency is shown in Table 2. SUVmax had an AUC of 0.828 with 95% confidence interval (CI) 0.739–0.917. SAD had an AUC of 0.729 with 95% CI 0.619–0.839. The AUC (95%CI), sensitivity, and specificity of the Int-Score were 0.872 (0.797–0.946), 0.895, and 0.625, respectively. The bar chart was used to intuitively display the discrimination performance of Int-Score, as shown in Fig. S6 (Appendix A-2). There was no significant difference in ROC curves of Int-Score (DeLong test, *p* = 0.836).

**Table 2:** Performance of models

Model	Cutoff	Training cohort					Testing cohort				
		AUC	95% CI	Accuracy	Specificity	Sensitivity	AUC	95% CI	Accuracy	Specificity	Sensitivity
SAD	*1.000*	0.697	0.622–0.773	0. 659	0.719	0.461	0.729	0.619–0.839	0.513	0.750	0.500
SUVmax	*2.500*	0.797	0.734–0.860	0.719	0.573	0.876	0.828	0.739–0.917	0.718	0.575	0.868
Rad-Score	− 0.044	0.849	0.796–0.903	0.768	0.802	0.730	0.828	0.782–0.919	0.769	0.725	0.816
Int-Score	0.331	0.881	0.834–0.928	0.789	0.688	0.899	0.872	0.797–0.946	0.756	0.625	0.895

The values in italics indicate the cut-off values used in the diagnosis process

*AUC*, the area under receiver operating characteristic (ROC) curves; *CI*, confidence interval

*SUVmax ≥ 2.5 or SAD ≥ 1.0 cm to be malignant LNs, which are usually used as reference standards in our diagnosis process

#### Calibration

Calibration curve is presented in Fig. S8 (Appendix A-2). The Hosmer-Lemeshow test showed no significant difference (*p* > 0.05) in the training cohort, demonstrating a good fit.

#### Clinical application

**Fig. 3:**
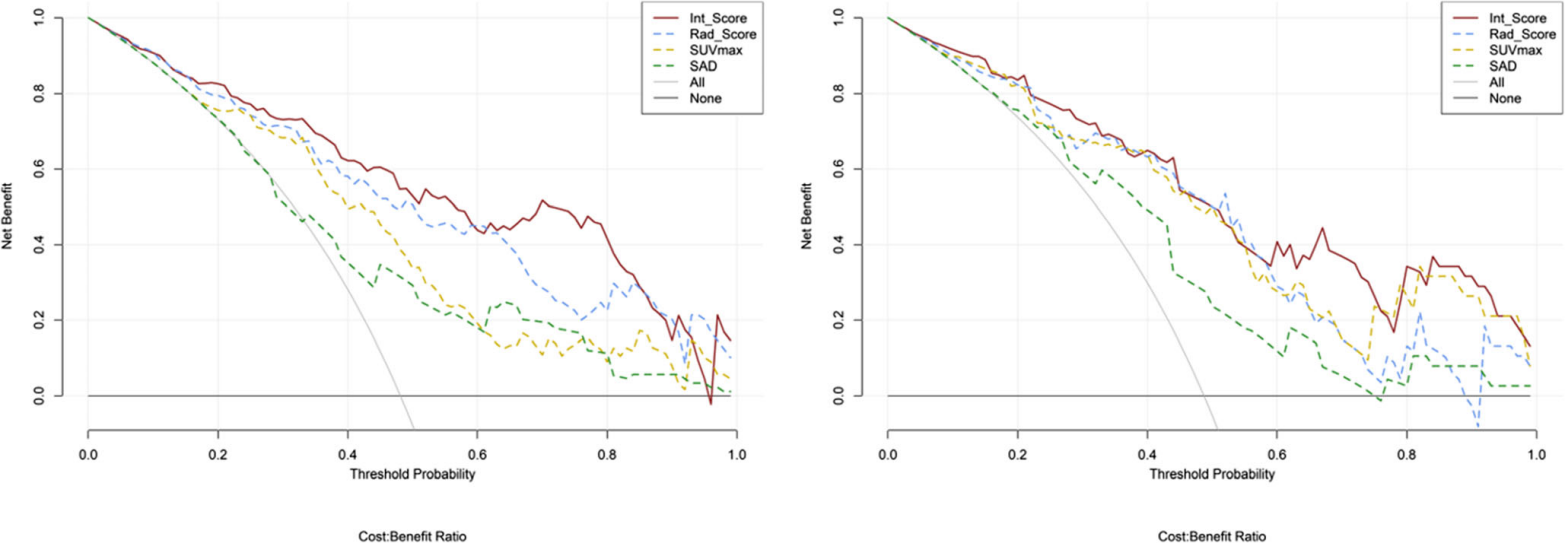
DCA for SUVmax, SAD, Rad-Score, and Int-Score in training (left) and testing (right) cohorts. The y-axis measures the net benefit. The y-axis measures the threshold probability. The solid red curve represents Int-Score. The blue dotted curve represents Rad-score. The yellow dotted curve represents SUVmax. The green dotted curve represents a short-axis diameter (SAD). The gray curve represents the assumption that all LN metastases. The black line represents the assumption that no LN metastases. The net benefit [40] was calculated by subtracting the proportion of all LNs, which are false positive from the proportion which is true positive, weighting by the relative harm of forgoing treatment compared with the negative consequences of unnecessary treatment

DCA is presented in Fig. 3. DCA showed that using SUVmax, SAD, Rad-Score, and Int-Score increases more benefit than the treat all project or the treat none project if the threshold probability of a patient or doctor is ˃ 10%. Compared to the other methods, Int-Score had a higher net benefit. As well as little overlaps within a range from 0.1 to 1.0, the curve of Int-Score is always at the top right.

## Discussion

In the present research, a radiomics model (Rad-Score) based on CT which aimed to noninvasively predict LN status was established, compared to, and combined with SUVmax (functional information) and SAD (measurable anatomical information), after which a PET/CT nomogram was built and validated. The PET/CT nomogram, here we called Int-Score, provides a more accurate preoperative estimation of LN status in patients with NSCLC, and, in turn, may guide N staging and further clinical decisions.

PET/CT is a widely used non-invasive imaging tool for evaluating the TNM stage in NSCLC patients. When using traditional diagnostic procedures, we comprehensively judge LN status by measuring SAD and attenuation, observing morphology and edges on CT image, and measuring SUV on PET image. Yet, artificially judging the change in density and evaluating the morphological edge of the lesion, many subjective outcomes would inevitably appear and a lot of information that cannot be visually perceived may be missed. In this study, we quantitatively extracted the information that is difficult to be visually perceived of LNs using radiomics, and accurately described the attenuation variation and shape difference of LNs [21, 22, 35], thus avoiding the one-sidedness of subjective judgments and visual differences to some extent. By using a nomogram (Int-Score) that incorporates the function information (SUVmax) and structural information (radiomics features), we provided a scale for the preoperative estimation of LN status. Previously, Billé A et al [16] suggested an AUC for the ratio of LN to primary tumor SUVmax multiplied by the maximal diameter of tumor, the ratio of LN to primary tumor SUVmax, and SUVmax of 0.709, 0.590, and 0.673, respectively. In this study, the Int-Score showed moderate improvement of the efficiency with AUC of 0.881 (95% CI, 0.834–0.928) in the training cohort and AUC of 0.872 (95% CI, 0.797–0.946) in the testing cohort. Moreover, compared with other studies [35], which examined texture features and multi-resolution histograms from ^18^F-FDG PET/CT images, Int-Score had better diagnostic efficacy. In addition, more detailed radiomics features were extracted to avoid the deviation of results caused by incomplete information. Previously, Bayanati H et al [36] combined three texture features and three shape-based features to predict LN metastasis with an accuracy of 0.71 and AUC of 0.87. By contrast, we examined the generalization and diagnostic efficacy of the model with an independent validation group, incorporating metabolic information, and accurately assessing the global morphology of focal using 3D VOI.

In this study, 20 patients of NSCLC with N2 were evaluated as clinical N1 or even N0 and underwent surgical resection. The sensitivity of PET/CT nomogram for N2 disease was 85% (17/20). Endobronchial ultrasound-guided transbronchial needle aspiration (EBUS-TBNA) is an invasive diagnostic tool for N2 diseases, which also can help improve the preoperative staging of these patients [37], but the puncture path is often restricted by anatomical location. Therefore, PET/CT nomogram seems to be more flexible and non-invasive in clinical use than minimally invasive EBUS-TBNA staging for patients with uncertain N2.

This study also has a few limitations. Firstly, the sample size was relatively small for radiomics analysis; however, we used a sample size estimation method [38] to prove that the testing cohort exceeded the minimum sample size required. A future multicenter study with an external validation dataset is necessary to improve and generalize our model. Secondly, there was some bias in the selection of LNs, and we may choose false-positive LNs with higher SUVmax, leading to a high false-positive rate in the overall data. Nevertheless, no significant differences were found in the distribution of SUVmax and SAD values between training and testing cohorts. Thirdly, our study did not include clinical information, such as CEA, CA125, and CA153 levels. Yang X et al [39] found that these clinical parameters were not independent predictors for LN metastasis; yet, a comprehensive assessment is still needed for clinical decisions.

## Conclusion

This study analyzed radiomics features extracted from LN CT imaging, and also developed and validated a PET/CT nomogram, which incorporated Rad-score and SUVmax for preoperative estimation of N staging in NSCLC. The PET/CT nomogram, which is a non-invasive predictive tool that improves the diagnostic accuracy, specificity, and sensitivity of N staging compared to SUVmax and SAD alone, can be used to better assist clinicians in making individual treatment decisions.

## Supplementary information

ESM 1 (DOCX 107408 kb)

## Abbreviations

ACC: Accuracy
AUC: Area under the curve
CA125: Carbohydrate antigen 125
CA153: Carbohydrate antigen 153
CEA: Carcinoembryonic antigen
CI: Confidence interval
CT: Computerized tomography
GLCM: Gray level co-occurrence matrix
GLDM: Gray level dependence matrix
GLRLM: Gray level run length matrix
GLSZM: Gray level size zone matrix
LASSO: The least absolute shrinkage and selection operator
LN: Lymph node
mRMR: Minimum-Redundancy Maximum-Relevance
NGTM: Neighboring gray-tone difference matrix
NSCLC: Non-small cell lung cancer
PET/CT: Positron-emission tomography/computed tomography
ROC: Receiver operating characteristic
ROI: Region of interest
SA: Section area
SAD: Short-axis diameter
SUV: Standard uptake value
SUVmax: Maximal standard uptake value
VOI: Volume of interest

## References

[1] Sakao Y, Okumura S, Mingyon M, Uehara H, Ishikawa Y, Nakagawa K (2011). The impact of superior mediastinal lymph node metastases on prognosis in non-small cell lung cancer located in the right middle lobe. J Thorac Oncol.

[2] Zhao Y, Li G, Zheng D (2017). The prognostic value of lymph node ratio and log odds of positive lymph nodes in patients with lung adenocarcinoma. J Thorac Cardiovasc Surg.

[3] Isaka M, Kojima H, Takahashi S, Omae K, Ohde Y (2018). Risk factors for local recurrence after lobectomy and lymph node dissection in patients with non-small cell lung cancer: implications for adjuvant therapy. Lung Cancer.

[4] Wang S, Zhou W, Zhang H, Zhao M, Chen X (2014). Analysis of predictive factors for postoperative survival for non small cell lung carcinoma patients with unexpected mediastinal lymph nodes metastasis. Thorac Cardiovasc Surg.

[5] Planchard D, Popat S, Kerr K (2018). Metastatic non-small cell lung cancer: ESMO clinical practice guidelines for diagnosis, treatment and follow-up. Ann Oncol.

[6] Boffa D, Fernandez FG, Kim S (2017). Surgically managed clinical stage IIIA-clinical N2 lung cancer in the Society of Thoracic Surgeons database. Ann Thorac Surg.

[7] Bilfinger T, Keresztes R, Albano D, Nemesure B (2016). Five-year survival among stage IIIA lung cancer patients receiving two different treatment modalities. Med Sci Monit.

[8] Bueno R, Richards WG, Swanson SJ (2000). Nodal stage after induction therapy for stage IIIA lung cancer determines patient survival. Ann Thorac Surg.

[9] Betticher DC, Hsu Schmitz SF, Totsch M (2003). Mediastinal lymph node clearance after docetaxel-cisplatin neoadjuvant chemotherapy is prognostic of survival in patients with stage IIIA pN2 non-small-cell lung cancer: a multicenter phase II trial. J Clin Oncol.

[10] Lorent N, De Leyn P, Lievens Y (2004). Long-term survival of surgically staged IIIA-N2 non-small-cell lung cancer treated with surgical combined modality approach: analysis of a 7-year prospective experience. Ann Oncol.

[11] Petersen H, Holdgaard PC, Madsen PH (2016). FDG PET/CT in cancer: comparison of actual use with literature-based recommendations. Eur J Nucl Med Mol Imaging.

[12] Fletcher JW, Djulbegovic B, Soares HP (2008). Recommendations on the use of 18F-FDG PET in oncology. J Nucl Med.

[13] Nambu A, Kato S, Sato Y (2009). Relationship between maximum standardized uptake value (SUVmax) of lung cancer and lymph node metastasis on FDG-PET. Ann Nucl Med.

[14] Vassallo P, Edel G, Roos N, Naguib A, Peters PE (1993). In-vitro high-resolution ultrasonography of benign and malignant lymph nodes. A sonographic-pathologic correlation. Invest Radiol.

[15] Flechsig P, Kratochwil C, Schwartz LH (2014). Quantitative volumetric CT-histogram analysis in N-staging of 18F-FDG-equivocal patients with lung cancer. J Nucl Med.

[16] Bille A, Pelosi E, Skanjeti A (2009). Preoperative intrathoracic lymph node staging in patients with non-small-cell lung cancer: accuracy of integrated positron emission tomography and computed tomography. Eur J Cardiothorac Surg.

[17] Xu N, Wang M, Zhu Z, Zhang Y, Jiao Y, Fang W (2014). Integrated positron emission tomography and computed tomography in preoperative lymph node staging of non-small cell lung cancer. Chin Med J (Engl).

[18] Lee BE, von Haag D, Lown T, Lau D, Calhoun R, Follette D (2007). Advances in positron emission tomography technology have increased the need for surgical staging in non-small cell lung cancer. J Thorac Cardiovasc Surg.

[19] Kiyono K, Sone S, Sakai F (1988). The number and size of normal mediastinal lymph nodes: a postmortem study. AJR Am J Roentgenol.

[20] Glazer GM, Gross BH, Quint LE, Francis IR, Bookstein FL, Orringer MB (1985). Normal mediastinal lymph nodes: number and size according to American Thoracic Society mapping. AJR Am J Roentgenol.

[21] Gillies RJ, Kinahan PE, Hricak H (2016). Radiomics: images are more than pictures, they are data. Radiology.

[22] Lambin P, Leijenaar RTH, Deist TM (2017). Radiomics: the bridge between medical imaging and personalized medicine. Nat Rev Clin Oncol.

[23] Wu S, Zheng J, Li Y (2017). A radiomics nomogram for the preoperative prediction of lymph node metastasis in bladder cancer. Clin Cancer Res.

[24] Dong Y, Feng Q, Yang W et al (2017) Preoperative prediction of sentinel lymph node metastasis in breast cancer based on radiomics of T2-weighted fat-suppression and diffusion-weighted MRI. Eur Radiol 10.1007/s00330-017-5005-7 28828635

[25] Huang YQ, Liang CH, He L (2016). Development and validation of a radiomics nomogram for preoperative prediction of lymph node metastasis in colorectal cancer. J Clin Oncol.

[26] Zhong Y, Yuan M, Zhang T, Zhang YD, Li H, Yu TF (2018). Radiomics approach to prediction of occult mediastinal lymph node metastasis of lung adenocarcinoma. AJR Am J Roentgenol.

[27] He Y, Kozaki K, Karpanen T (2002). Suppression of tumor lymphangiogenesis and lymph node metastasis by blocking vascular endothelial growth factor receptor 3 signaling. J Natl Cancer Inst.

[28] Hoshida T, Isaka N, Hagendoorn J (2006). Imaging steps of lymphatic metastasis reveals that vascular endothelial growth factor-C increases metastasis by increasing delivery of cancer cells to lymph nodes: therapeutic implications. Cancer Res.

[29] Tanaka T, Imamura T, Yoneda M (2016). Enhancement of active MMP release and invasive activity of lymph node metastatic tongue cancer cells by elevated signaling via the TNF-alpha-TNFR1-NF-kappaB pathway and a possible involvement of angiopoietin-like 4 in lung metastasis. Int J Oncol.

[30] Asamura H, Chansky K, Crowley J (2015). The International Association for the Study of Lung Cancer Lung Cancer Staging Project: proposals for the revision of the N descriptors in the forthcoming 8th edition of the TNM classification for lung cancer. J Thorac Oncol.

[31] Zwanenburg A, Leger S, Vallieres M, Lock S. Image biomarker standardisation initiative. Available via https://arxiv.org/pdf/1612.07003.pdf

[32] Hellwig D, Graeter TP, Ukena D (2007). 18F-FDG PET for mediastinal staging of lung cancer: which SUV threshold makes sense?. J Nucl Med.

[33] Ding C, Peng H (2003) Minimum redundancy feature selection from microarray gene expression data. in Computational Systems Bioinformatics. CSB2003. Proceedings of the 2003 IEEE Bioinformatics Conference. CSB2003 10.1142/s0219720005001004 15852500

[34] Sauerbrei W, Royston P, Binder H (2007). Selection of important variables and determination of functional form for continuous predictors in multivariable model building. Stat Med.

[35] Gao X, Chu C, Li Y (2015). The method and efficacy of support vector machine classifiers based on texture features and multi-resolution histogram from (18)F-FDG PET-CT images for the evaluation of mediastinal lymph nodes in patients with lung cancer. Eur J Radiol.

[36] Bayanati HE, Thornhill R, Souza CA (2015). Quantitative CT texture and shape analysis: can it differentiate benign and malignant mediastinal lymph nodes in patients with primary lung cancer?. Eur Radiol.

[37] Vial MR, O'Connell OJ, Grosu HB (2018). Diagnostic performance of endobronchial ultrasound-guided mediastinal lymph node sampling in early stage non-small cell lung cancer: a prospective study. Respirology.

[38] Chow SC, Wang H, Shao J (2007) Sample size calculations in clinical research, 2nd edn. Taylor & Francis

[39] Yang X, Pan X, Liu H (2018). A new approach to predict lymph node metastasis in solid lung adenocarcinoma: a radiomics nomogram. J Thorac Dis.

[40] Vickers AJ, Cronin AM, Elkin EB, Gonen M (2008). Extensions to decision curve analysis, a novel method for evaluating diagnostic tests, prediction models and molecular markers. BMC Med Inform Decis Mak.

