# Supplementary material for: A PET/CT nomogram incorporating SUVmax and CT radiomics for preoperative nodal staging in non-small cell lung cancer
Source: Eur Radiol. 2021 Feb 9;31(8):6030–8. doi: 10.1007/s00330-020-07624-9 (PMC8270849; doi:10.1007/s00330-020-07624-9)
Supplement: Supplementary file 1 — (DOCX 107408 kb) [file 330_2020_7624_MOESM1_ESM.docx]

**Appendix A.** Supplementary information for Int-Score

**1.VOI Selection and Segmentation**

With the progress of the medical industry and the popularity of precision medicine, we discovered that NSCLC patients with different N stages might have different prognoses. Previously, Chen W *et al* indicated that the five-year survival rate of patients in stages N0, N1, and N2 is 76.1%, 53.4%, and 26.3%, respectively [1]. Additionally, Hanagiri T *et al* [2] and Yoo C *et al* [3] stratified and analyzed the number of involved LNs in III-N2 NSCLC patients and found different prognosis, suggesting that surgery may benefit patients with one or two LNs. Besides, some other studies recommended surgical treatment for patients with single-station N2 due to a better prognosis [4-7]. All these data suggest that preoperative N staging, as well as metastatic LNs, may have an important determinant factor for the prognosis and individualized treatment decision in NCSLC patients. In this study, we selected LNs as VOIs rather than primary tumors and offered a solution for the quantitative evaluation of the LN metastasis.

The VOIs were semi-automatically segmented using imaging post-processing software (Intellispace Discovery, Philips Healthcare) by three radiologists. Firstly, the CT images were adjusted to soft tissue windows (Window Width: 300, Window Level: L40) in order to clearly observe the LNs boundaries. Secondly, a junior radiologist (2 years diagnosis experience of chest CT), who selected LNs according to the inclusion criteria, and recorded the anatomical location of LNs and primary tumors (**Table 1 and Table S3**). To avoid the subjectivity of the VOI boundary and volume as much as possible, another senior radiologist (5 years diagnosis experience of chest CT), who was blinded to the pathological results, created VOIs using a 3D semi-automatic creation tool on the largest cross-sectional area of LNs, and modified the contours to completely contain the LNs using a 3D semi-automatic edition tool and excluded other issues (such as blood vessels, trachea, and healthy lung tissues or organs) layer by layer using a 2D manual edition tool. Then, to ensure the robustness of the radiomics feature, another senior radiologist (7 years diagnosis experience of chest CT), who had no prior knowledge about the pathological result, reviewed the VOI one by one. The final VOIs were generated for further radiomics analysis. All segmentation tools used the same Philips proprietary algorithm (Simplicit; Medisys, Philips Healthcare).

**2.Figures of Int-Score**

**Figure S4.**

PET/CT Nomogram was used to predict LN status in patients with NSCLC. The PET/CT nomogram was developed in the training cohort, with the Rad-Score and SUVmax incorporated using multivariate logistic regression analysis. The range of Rad-Score and SUVmax were -6~12 and 0~18, respectively. The scores of Rad-Score and SUVmax are the corresponding numbers on the points scale. The scores of Rad-Score and SUVmax were added up to get the total scores. The probability of LN metastasis is the corresponding numbers on the lower probability of the Invasion scale.

**Figure S5.**

ROC curves of SUVmax, SAD, Rad-Score, and Int-Score in training and testing cohort. The receiver operating characteristic curve is based on the confusion matrix. The 95% confidence interval (CI), which is calculated at a predetermined level of significance (α=0.005), shows the degree to which the true value of LN metastasis has a certain probability of falling around the predicted value, and it gives the confidence degree of the predicted value of LN metastasis. The y-axis represents the sensitivity of the predicted LN metastasis; the x-axis represents the 1-specificity of predicted LN metastasis. The solid grey line represents a stochastic model without diagnosis value. The solid red curve represents the performance of Int-Score; the blue dotted curve represents Rad-score; the yellow dotted curve represents SUVmax; the green dotted curve represents short-axis diameter (SAD).

**Figure S6**.

Bar chart of Int-Score in training (upper) and testing (lower) cohorts. The y-axis refers to the Int-Score minus the optimal cutoff value (i.e., Int-Score - 0.331). Upper and lower bars refer to the predicted malignant and benign LNs, respectively. Green and red bars refer to actual malignant and benign LN, respectively.

**Figure S7.**

Representative PET/CT images showing LNs features in the negative and the positive groups. **(A, F)** CT images; **(B, G)** PET images; **(C, H)** fusion images of PET and CT; **(D, I)** segmented images; **(E, J)** bronchovascular reconstruction images with VOIs. The upper row displays a PET false-positive case in the LN (-) group (SUVmax, SAD, Rad-Score, and Int-Score of LN01 of 4.698,1.3 cm, -0.514 and -0.094, respectively) and in LN02 (4.698, 0.8 cm, -0.722 and -0.330, respectively). The lower row displays a PET false-negative case in LN (+) group; SUVmax, SAD, Rad-Score, and Int-Score of LN03 were 2.051,0.5 cm,1.507 and 1.099, respectively.

**Figure S8.**

Calibration Curves of Int-Score in training (left) and testing (right) cohorts. Calibration curves depict the agreement between the predicted risks of lymph node (LN) metastasis and actual observed outcomes of LN metastasis. The y-axis represents the actual LN metastasis probability. The x-axis represents the predicted LN metastasis probability. The solid grey line represents a perfect prediction by an ideal model with an accuracy of 1. The red dotted curve represents the performance of Int-Score; a closer ﬁt to the solid grey line represents a better predictive ability.

**3.The redundancy of SAD in Int-Score**

In the present study, anatomical information (SAD), functional information (SUVmax), and radiomics features (Rad-Score), which were significantly different between LN (+) and LN (-) groups (**Table 1**), has been tried to use for PET/CT nomogram construction. However, the SAD effect on model optimization was low, showing a mild decline of AUC in both training and testing cohorts. This may be because the size of LNs is not a reliable parameter for the evaluation of metastatic LN involvement in patients with NSCLC [8-10]. SAD, the mean in LN (+) group and LN (-) group roughly equal to the study of Yang F *et al* [11] with the really radiological-pathological one-to-one matching of LNs, also revealed this truth with lower sensitivity (training cohort:0.461; testing cohort:0.500) and specificity (training cohort: 0.719; testing cohort:0.750) with a cutoff value of 10.0 mm [1] in this study. What is more, considered that the radiomics features include shape-based features and first-order histogram features, of which SAD was contained. Thus, in the Int-Score, SAD was removed due to its redundancy.

**4.The role of SUVmax in Int-Score**

Given the susceptibility to noise, the low spatial and anatomic resolution and the small size of segmented LNs, PET images alone may not be enough to efficiently extract the radiomics features of LNs [12, 13]. Thus, in this study, we adopted SUVmax, the most representative semi-quantitative data, which reflects the ability of the tissue to absorb ^18^F-FDG as the functional information of LNs. The conventional PET diagnostic criteria (SUVmax ≥2.5) [14-16] are usually considered for malignant LNs. SUVmax with a threshold of 2.5 in our study showed a sensitivity of 0.876 in the training cohort and 0.868 in the testing cohort compared with the new criterion—the ratio of section area (SA) of SUV≥2.5/ LN SA ≥ 1.0 [17]. Moreover, considering that the radiomics features did not include metabolic information, we contained SUVmax in Int-score. ROC curves and DCA curves demonstrated that Int-Score had better diagnostic performance and more clinical net beneﬁt for patients compared to Rad-Score and SUVmax alone. Although Rad-Score and SUVmax yielded the same AUC values of 0.828 in the testing cohort, and Rad-Score with an AUC of 0.835, which was slightly higher than SUVmax in the training cohort; they were not equivalent, but complementary. Rad-Score made up for the shortcomings of SUVmax with low specificity and reduced the false-positive rate of the diagnosis of LN metastasis, while SUVmax improved the sensitivity of Int-Score; the combination of these two could improve the overall effectiveness of the model. Therefore, consistent with previous studies [18, 19], the diagnostic efficacy of integrated PET/CT nomogram was better compared to CT radiomics and SUVmax alone.

**REFERENCES**

1. Chen W, Zhang C, Wang G, Yu Z,Liu H (2019) Feasibility of nodal classification for non-small cell lung cancer by merging current N categories with the number of involved lymph node stations. Thorac Cancer 10:1533-1543

2. Hanagiri T, Takenaka M, Oka S et al (2011) Clinical significance in the number of involved lymph nodes in patients that underwent surgery for pathological stage III-N2 non-small cell lung cancer. J Cardiothorac Surg 6:144

3. Yoo C, Yoon S, Lee DH et al (2015) Prognostic Significance of the Number of Metastatic pN2 Lymph Nodes in Stage IIIA-N2 Non-Small-Cell Lung Cancer After Curative Resection. Clin Lung Cancer 16:e203-12

4. Keller SM, Vangel MG, Wagner H et al (2004) Prolonged survival in patients with resected non-small cell lung cancer and single-level N2 disease. J Thorac Cardiovasc Surg 128:130-7

5. Maniwa T, Shintani Y, Okami J et al (2018) Upfront surgery in patients with clinical skip N2 lung cancer based on results of modern radiological examinations. J Thorac Dis 10:6828-6837

6. Inoue M, Sawabata N, Takeda S, Ohta M, Ohno Y,Maeda H (2004) Results of surgical intervention for p-stage IIIA (N2) non-small cell lung cancer: acceptable prognosis predicted by complete resection in patients with single N2 disease with primary tumor in the upper lobe. J Thorac Cardiovasc Surg 127:1100-6

7. Yoshino I, Yoshida S, Miyaoka E et al (2012) Surgical outcome of stage IIIA- cN2/pN2 non-small-cell lung cancer patients in Japanese lung cancer registry study in 2004. J Thorac Oncol 7:850-5

8. Prenzel KL, Monig SP, Sinning JM et al (2003) Lymph node size and metastatic infiltration in non-small cell lung cancer. Chest 123:463-7

9. Ikeda K, Nomori H, Mori T, Kobayashi H, Iwatani K,Yoshimoto K (2006) Size of metastatic and nonmetastatic mediastinal lymph nodes in non-small cell lung cancer. J Thorac Oncol 1:949-52

10. Caupena C, Costa R, Pérez-Ochoa F et al (2019) Nodal size ranking as a predictor of mediastinal involvement in clinical early-stage non-small cell lung cancer. Medicine (Baltimore) 98:e18208

11. Yang F, Dong J, Wang X, Fu X,Zhang T (2017) Non-small cell lung cancer: Spectral computed tomography quantitative parameters for preoperative diagnosis of metastatic lymph nodes. Eur J Radiol 89:129-135

12. Gao X, Chu C, Li Y et al (2015) The method and efficacy of support vector machine classifiers based on texture features and multi-resolution histogram from (18)F-FDG PET-CT images for the evaluation of mediastinal lymph nodes in patients with lung cancer. Eur J Radiol 84:312-7

13. Mayerhoefer ME, Materka A, Langs G et al (2020) Introduction to Radiomics. J Nucl Med 61:488-495

14. Hellwig D, Graeter TP, Ukena D et al (2007) 18F-FDG PET for mediastinal staging of lung cancer: which SUV threshold makes sense? J Nucl Med 48:1761-6

15. Bryant AS, Cerfolio RJ, Klemm KM,Ojha B (2006) Maximum standard uptake value of mediastinal lymph nodes on integrated FDG-PET-CT predicts pathology in patients with non-small cell lung cancer. Ann Thorac Surg 82:417-22; discussion 422-3

16. Okereke IC, Gangadharan SP, Kent MS, Nicotera SP, Shen C,DeCamp MM (2009) Standard uptake value predicts survival in non-small cell lung cancer. Ann Thorac Surg 88:911-5; discussion 915-6

17. Saito Y, Imai K, Ishiyama K et al (2017) New PET/CT criterion for nodal staging in non-small cell lung cancer: measurement of the ratio of section area of standard uptake values >/=2.5/lymph node section area. Gen Thorac Cardiovasc Surg 65:350-357

18. Lardinois D, Weder W, Hany TF et al (2003) Staging of non-small-cell lung cancer with integrated positron-emission tomography and computed tomography. N Engl J Med 348:2500-7

19. Halpern BS, Schiepers C, Weber WA et al (2005) Presurgical staging of non-small cell lung cancer: positron emission tomography, integrated positron emission tomography/CT, and software image fusion. Chest 128:2289-97

**Appendix B.** Details of Extracted Radiomics Features

**1. First-order statistics (N=18)**

- Interquartile Range
- Skewness
- Uniformity
- Median
- Energy
- Robust Mean Absolute Deviation
- Mean Absolute Deviation
- Total Energy
- Maximum
- Root Mean Squared
- 90Percentile
- Minimum
- Entropy
- Range
- Variance
- 10Percentile
- Kurtosis
- Mean

**2. High-order statistics**

**(1) Gray Level Cooccurrence Matrix (N=22)**

- Joint Average
- Joint Entropy
- Cluster Shade
- Maximum Probability
- Idmn
- Joint Energy
- Contrast
- Difference Entropy
- Inverse Variance
- Difference Variance
- Idn
- Idm
- Correlation
- Autocorrelation
- Sum Entropy
- Sum Squares
- Cluster Prominence
- Imc2
- Imc1
- Difference Average
- Id
- Cluster Tendency

**(2) Gray Level Run Length Matrix (N=16)**

- Gray Level Variance
- Zone Variance
- Gray Level Non-Uniformity Normalized
- Size Zone Non-Uniformity Normalized
- Size Zone Non-Uniformity
- Gray Level Non-Uniformity
- Large Area Emphasis
- Small Area High Gray Level Emphasis
- Zone Percentage
- Large Area Low Gray Level Emphasis
- Large Area High Gray Level Emphasis
- High Gray Level Zone Emphasis
- Small Area Emphasis
- Low Gray Level Zone Emphasis
- Zone Entropy
- Small Area Low Gray Level Emphasis

**(3) Gray Level Size Zone Matrix (N=16)**

- Short Run Low Gray Level Emphasis
- Gray Level Variance
- Low Gray Level Run Emphasis
- Gray Level Non-Uniformity Normalized
- Run Variance
- Gray Level Non-Uniformity
- Long Run Emphasis
- Short Run High Gray Level Emphasis
- Run Length Non-Uniformity
- Short Run Emphasis
- Long Run High Gray Level Emphasis
- Run Percentage
- Long Run Low Gray Level Emphasis
- Run Entropy
- High Gray Level Run Emphasis
- Run Length Non-Uniformity Normalized

**(4) Neighboring Gray Tone Difference Matrix (N=5)**

- Coarseness
- Complexity
- Strength
- Contrast
- Busyness

**(5) Gray Level Dependence Matrix (N=14)**

- Gray Level Variance
- High Gray Level Emphasis
- Dependence Entropy
- Dependence Non-Uniformity
- Gray Level Non-Uniformity
- Small Dependence Emphasis
- Small Dependence High Gray Level Emphasis
- Dependence Non-Uniformity Normalized
- Large Dependence Emphasis
- Large Dependence Low Gray Level Emphasis
- Dependence Variance
- Large Dependence High Gray Level Emphasis
- Small Dependence Low Gray Level Emphasis
- Low Gray Level Emphasis

**3. Shape-based features (N=16)**

- Spherical Disproportion
- Compactness2
- Maximum 2D Diameter Slice
- Sphericity
- Minor Axis
- Compactness1
- Elongation
- Surface Volume Ratio
- Volume
- Maximum 3D Diameter
- Major Axis
- Surface Area
- Flatness
- Least Axis
- Maximum 2D Diameter Column
- Maximum 2D Diameter Row

**Appendix C.** Details of Rad-Score

**1.Details of Features selection**

**Figure S1.**

The correlation graphs of LASSO logistic regression for radiomics feature selection. Y-coordinate represents the binomial deviance (left plot) and the LASSO coefficients of 30 features selected by the Minimum Redundancy Maximum Relevance (mRMR) algorithm (right plot). Lower X-coordinate indicates the log (λ) that was chosen (1-SE criteria) according to 10-fold cross-validation. Upper X-coordinate represents the average number of features in the optimized subset. Dotted vertical lines (left plot) were drawn at the optimal values using the minimum criteria and the tuning parameter (λ) standard error of the minimum criteria (the 1-SE criteria). The dotted vertical line (right plot) was drawn at the number of features in the optimal subset selected using 10-fold cross-validation, where optimal λ (1.0220) resulted in 14 nonzero coefficients features and corresponded log(λ) value of 0.0096.

**Figure S2**.

The coefficients of selected features were obtained by multiple logistic regression analyses.

**Figure S3.**

Box plot of the radiomics model. Y-axis indicates the radiomics score (Rad-Score); X-axis indicates the benign or malignant classification. The black horizontal line represents the cutoff value of (Rad-Score) with -0.044 in training (left), which also applied in the testing cohort (right), LNs with Rad-scores higher than -0.044 are classified as 1; LNs with Rad-scores lower than -0.044 are classified as 0. Wilcoxon rank-sum test was no significant difference in the clinical characteristics between benign LNs (Label 0) and malignant LNs (Label 1).

**2.Result of Rad-Score**

A total of 1472 features were reduced to 30 strongest features by mRMR algorithm, and to 14 nonzero coefﬁcients predictors by the LASSO method in the training cohort (**Table S2**). Calculation of the Rad-Score was performed using the formula:

Rad-Score=0.332 × Wavelet_LLL-GLCM-Inverse Variance

+0.787 × Log_sigma_1_0_mm_3D-NGTDM-Busyness

+(-0.021) × Exponential-GLSZM-Small Area Low Gray Level Emphasis

+(-0.27) × Wavelet_HHL-NGTDM-Strength

+(-0.004) × Exponential-First Order-Variance

+0.42 × Wavelet_HLH-GLSZM-Size Zone Non-Uniformity Normalized

+(-0.466) × Log_sigma_1_0_mm_3D-Firstorder-Median

+0.064 × Original-GLSZM-Large Area High Gray Level Emphasis

+(-0.337) × Wavelet_HHL-GLCM-Correlation

+(-0.154) × Wavelet_LHL-First Order-Median

+(-0.797) × Gradient-GLCM-Inverse Variance

+0.084 × Log_sigma_3_0_mm_3D-Firstorder-Skewness

+(-0.089) × Wavelet_LHH-Glcm-Cluster Shade

+0.185 × Log_sigma_3_0_mm_3D-GLRLM-Run Variance

+0.055

The thresholds of the binomials are -0.044 in the training cohort applied in the testing cohort. Malignant LNs had higher Rad-Score values in both training and testing cohorts.

The Rad-Score had an AUC (95%CI) of 0.849 (0.796-0.903) in the training cohort with sensitivity of 0.730, and specificity of 0.802. With a threshold of -0.044, the Rad-Score demonstrated good diagnostic performance in the testing cohort with AUC (95%CI) of 0.828 (0.782-0.919), sensitivity of 0.816, and specificity of 0.725.

The results of this study also show that image information of lesions with small size, fine structure, and various shapes, which may be challenging to assess using the conventional method, can be effectively transformed into quantitative data using radiomics. Similarly, radiomics based on LNs (Rad-Score) with AUC of 0.849 in the training cohort and 0.828 in the testing cohort can be used to predict N-staging, which is better than the classifier based on only texture features and multi-resolution histogram from ^18^F-FDG PET/CT images [1]. Moreover, 14 radiomics features selected by mRMR algorithm and LASSO method, including *Median,* *Small Area Low Gray Level Emphasis,* *Large Area High Gray Level Emphasis* and *Strength* revealed that higher CT value might be associated with the malignancy, which is consistent with Flechsig P *et al* [2-4]; the CT value between 20-40Hu suggests an 88% chance of malignancy. Of course, this conclusion may be based on the earlier clinical stage of patients and the selected LNs have neither obvious necrosis nor calcification in our study. Interestingly enough, the *Variance*, *skewness*, *Cluster Shade*, and *Busyness* are consistent with previous studies [5-7], which indicated that the more heterogeneous images are linked with, the higher probability of metastatic LN. Besides, the morphological parameter of LN was also considered to be related to metastasis [7, 8]. Yet, none of the shape-based features was selected in our study due to its redundancy. Fusco R *et al* [8] found *compactness [LN surface area/LN volume]* as a shape feature was one of the best parameters for identifying metastatic LNs on breast MRI with 3D VOI. Moreover, they calculated that only *circularity [(volume of the sphere with average LN radius)/(LN volume)]* showed no significant difference between benign LNs group and malignant LNs group. On the contrary, Bayanati H *et al* [7] found that the shape factors directly related to LN roundness were significantly elevated in the malignant LNs group on chest CT. Given that Fusco R *et al* [8] only analyzed the shape-based features, but did not extract first-order histogram features, higher-order texture features, and transformed features. Bayanati H *et al* [7] segmented a 2D ROI which did not accurately assess the overall shape of the lesion, lacked a validation using an independent group to assess the generalizability and performance of the classifier，and also deficiency of analysis and discussion of function information. Furthermore, the robustness and repeatability of the shape feature can be easily affected by the segmentation algorithm and the individual differences of doctors in identifying lesion margins. Hence, the shape-based features of LN produced by VOI and its importance in relation to other types of features need to be further studied.

**REFERENCES**

1. Gao X, Chu C, Li Y et al (2015) The method and efficacy of support vector machine classifiers based on texture features and multi-resolution histogram from (18)F-FDG PET-CT images for the evaluation of mediastinal lymph nodes in patients with lung cancer. Eur J Radiol 84:312-7

2. Flechsig P, Kratochwil C, Schwartz LH et al (2014) Quantitative Volumetric CT-Histogram Analysis in N-Staging of 18F-FDG-Equivocal Patients with Lung Cancer. Journal of Nuclear Medicine 55:559-564

3. Flechsig P, Frank P, Kratochwil C et al (2017) Radiomic Analysis using Density Threshold for FDG-PET/CT-Based N-Staging in Lung Cancer Patients. Mol Imaging Biol 19:315-322

4. Giesel F, Schneider F, Kratochwil C et al (2017) Correlation Between SUVmax and CT Radiomic Analysis Using Lymph Node Density in PET/CT-Based Lymph Node Staging. J. Nucl. Med. 58:282-287

5. Brown G, Richards CJ, Bourne MW et al (2003) Morphologic predictors of lymph node status in rectal cancer with use of high-spatial-resolution MR imaging with histopathologic comparison. Radiology 227:371-7

6. Andersen MB, Harders SW, Ganeshan B, Thygesen J, Madsen HHT,Rasmussen F (2016) CT texture analysis can help differentiate between malignant and benign lymph nodes in the mediastinum in patients suspected for lung cancer. Acta Radiol. 3:669-676

7. Bayanati H, E. Thornhill R, Souza CA et al (2015) Quantitative CT texture and shape analysis: Can it differentiate benign and malignant mediastinal lymph nodes in patients with primary lung cancer? European Radiology 25:480-487

8. Fusco R, Sansone M, Granata V et al (2018) Use of Quantitative Morphological and Functional Features for Assessment of Axillary Lymph Node in Breast Dynamic Contrast-Enhanced Magnetic Resonance Imaging. Biomed Res Int 2018:2610801

**Table S1** The stages of this study cohort (8^th^ Edition of the TNM Classification for Lung Cancer)

| **Stages** | **pN0** | **pN1** | **pN2** | **pN3** | **p or cM1a and any N** | **cM1b and any N** | **cM1c and any N** |
| --- | --- | --- | --- | --- | --- | --- | --- |
| **T1a** | pIA1(n=20) | pIIB(n=1) | pIIIA(n=0) | cIIIB(n=0) | cIVA(n=0) | cIVA(n=0) | cIVB(n=0) |
| **T1b** | pIA2(n=11) | pIIB(n=4) | pIIIA(n=3) | cIIIB(n=1) | cIVA(n=0) | cIVA(n=0) | cIVB(n=0) |
| **T1c** | pIA3(n=7) | pIIB(n=5) | pIIIA(n=4) | cIIIB(n=1) | cIVA(n=0) | cIVA(n=0) | cIVB(n=2) |
| **T2a** | pIB(n=20) | pIIB(n=9) | pIIIA(n=6) | cIIIB(n=1) | cIVA(n=0) | cIVA(n=0) | cIVB(n=2) |
| **T2b** | pIIA(n=2) | pIIB(n=3) | pIIIA(n=2) | cIIIB(n=1) | cIVA(n=0) | cIVA(n=0) | cIVB(n=0) |
| **T3** | pIIB(n=1) | pIIIA(n=4) | pIIIB(n=4) | cIIIC(n=3) | p&cIVA(n=2) | cIVA(n=1) | cIVB(n=1) |
| **T4** | pIIIA(n=1) | pIIIA(n=1) | pIIIB(n=0) | cIIIC(n=0) | cIVA(n=0) | cIVA(n=0) | cIVB(n=0) |

Note:p: pathological; c: clinical.

Total: IA (n=38), IB (n=20), IIA (n=2), IIB (n=23), IIIA (n=22), IIIB (n=8), IIIC(n=3), IVA(n=3), IVB(n=5);

Surgery: pIA (n=38), pIB (n=20), pIIA (n=2), pIIB (n=23), pIIIA (n=22), pIIIB (n=4), pIVA(n=1);

Supra-clavicular LN biopsies: cIIIB (n=4), cIIIC(n=3), cIVA(n=2), cIVB(n=5).

**Table S2** Details of Selected Features in the Rad-Score

| Feature | Radiomic Group | Associated Filter | Description | Formula |
| --- | --- | --- | --- | --- |
| Median | First-order | log_sigma_1_0_mm_3D  wavelet_LHL | The median gray level intensity within the ROI. | - |
| Variance | First-order | exponential | Variance is the mean of the squared distances of each intensity value from the Mean value. This is a measure of the spread of the distribution about the mean. | $\mathrm{Variance}=\frac{1}{N_{p}}\sum_{i=1}^{N_{p}} \left( X\left( i \right)-\bar{X} \right)^{2}$ |
| Inverse Variance | GLCM | wavelet_LLL  gradient | Inverse-variance weighting is a method of aggregating two or more random variables to minimize the variance of the sum. | $Inverse Variance=\sum_{K=1}^{N_{g}-1} \frac{P_{x-y}\left( k \right)}{k^{2}}$ |
| Correlation | GLCM | wavelet_HHL | Correlation is a value between 0 (uncorrelated) and 1 (perfectly correlated), showing the linear dependency of gray level values to their respective voxels in the GLCM. | $\mathrm{Correlation}=\frac{\sum_{i=1}^{N_{g}} \sum_{j=1}^{N_{g}} p^{\left( i,j \right)ij-\mu_{x}\mu_{y}}}{\sigma_{x}\left( i \right)\sigma_{y}\left( j \right)}$ |
| Cluster Shade | GLCM | wavelet_LHH | Cluster Shade is a measure of the skewness and uniformity of the GLCM. A higher cluster shade implies greater asymmetry about the mean. | $Cluster Shade=\sum_{i=1}^{N_{g}} \sum_{j=1}^{N_{g}} \left( i+j-\mu_{x}-\mu_{y} \right)^{3}p\left( i,j \right)$ |
| Busyness | NGTDM | log_sigma_1_0_mm_3D | A measure of the change from a pixel to its neighbour. A high value for busyness indicates a ‘busy’ image, with rapid changes of intensity between pixels and its neighbourhood. | $\mathrm{Busyness}=\frac{\sum_{i=1}^{N_{g}} p_{i}S_{i}}{\sum_{i=1}^{N_{g}} \sum_{j=1}^{N_{g}} \vert iP_{i}-P_{j}\vert},whereP_{i}\neq0,P_{j}\neq0$ |
| Strength | NGTDM | wavelet_HHL | Strength is a measure of the primitives in an image. Its value is high when the primitives are easily defined and visible, i.e., an image with a slow change in intensity but more large coarse differences in gray level intensities. | $\mathrm{Strength}=\frac{\sum_{i=1}^{N_{g}} \sum_{j=1}^{N_{g}} \left( p_{i}+p_{j} \right)\left( i-j \right)^{2}}{\sum_{i=1}^{N_{g}} S_{i}},whereP_{i}\neq0,P_{j}\neq0$ |
| Small Area Low Gray Level Emphasis | GLSZM | exponential | SALGLE measures the proportion in the image of the joint distribution of smaller size zones with lower gray-level values. | $SALGE= \frac{\sum_{i=1}^{N_{g}} \sum_{j=1}^{N_{s}} \frac{\mathbf{P}\left( i,j \right)}{i^{2}j^{2}}}{N_{z}}$ |
| Size Zone Non- Uniformity Normalized | GLSZM | wavelet_HLH | SZNN measures the variability of size zone volumes throughout the image, with a lower value indicating more homogeneity among zone size volumes in the image. | $SZNN=\frac{\sum_{j=1}^{N_{s}} \left( \sum_{i=1}^{N_{g}} \mathbf{P}\left( i,j \right) \right)^{2}}{N_{z}^{2}}$ |
| Large Area High Gray Level Emphasis | GLSZM | Original | LAHGLE measures the proportion in the image of the joint distribution of larger size zones with higher gray-level values. | $LAHGLE=\frac{\sum_{i=1}^{N_{g}} \sum_{j=1}^{N_{s}} \mathbf{P}\left( i,j \right)i^{2}j^{2}}{N_{z}}$ |
| Run Variance | GLRLM | log_sigma_3_0_mm_3D | RV is a measure of the variance in runs for the run lengths. | $RV=\sum_{i=1}^{N_{g}} \sum_{j=1}^{N_{r}} {p\left( i,j\vert\theta\right)\left( j-\mu\right)}^{2}，here,\mu=\sum_{i=1}^{N_{g}} \sum_{j=1}^{N_{r}} p\left( i,j\vert\theta\right)j$ |
| Skewness | First-order | log_sigma_3_0_mm_3D_ | Skewness measures the asymmetry of the distribution of values about the Mean value. Depending on where the tail is elongated, and the mass of the distribution is concentrated, this value can be positive or negative. | $skewness=\frac{\mu^{3}}{\sigma^{3}}=\frac{\frac{1}{N_{p}}\sum_{i=1}^{N_{p}} \left( \mathbf{X}\left( i \right)-\bar{X} \right)^{3}}{\left( \sqrt{\frac{1}{N_{p}}\sum_{i=1}^{N_{p}} \left( \mathbf{X}\left( i \right)-\bar{X} \right)^{2}} \right)^{3}},\mathrm{where}\mu^{3} is the 3\mathrm{rd} central moment$ |

Note:

a) Wavelet: Wavelet filtering, yields 8 decompositions per level (all possible combinations of applying either a High or a Low pass filter in each of the three dimensions);
b) Log: Laplacian of Gaussian filter, edge enhancement filter. Emphasizes areas of gray level change, where sigma defines how coarse the emphasis texture should be. A low sigma emphasis on fine textures (change over a short distance), where a high sigma value emphasis coarse textures (gray level change over a large distance);
c) SquareRoot: Takes the square root of the absolute image intensities and scales them back to the original range. Negative values in the original image will be made negative again after application of filter;
d) Exponential: Takes the exponential, where filtered intensity is e^(absolute intensity). Values are scaled to the original range, and negative original values are made negative again after application of filter;
e) Gradient: Returns the magnitude of the local gradient;
f) LocalBinaryPattern3D: Computes the Local Binary Pattern in 3D using spherical harmonics;
g) First-order: statistics describe the distribution of voxel intensities within the image region defined by the mask through commonly used and basic metrics;
h) GLCM: Gray Level Co-occurrence Matrix;
i) NGTDM: Neighbouring Gray Tone Difference Matrix;
j) GLSZM: Gray Level Size Zone Matrix.

**Table S3 Details of lymph node stations**

|  | **Training cohort** | | |  | **Testing cohort** | | | **P^##^ value** |
| --- | --- | --- | --- | --- | --- | --- | --- | --- |
|  | **LN (+)**  **(N=89)** | **LN (-)**  **(N=96)** | **P value** |  | **LN (+)**  **(N=38)** | **LN (-)**  **(N=40)** | **P value** |  |
| **LN station/No. (%)** |  |  | 0.01 |  |  |  | 0.058 | 0.154 |
| **#1** | 10(3.80%) | 0(0.00%) |  |  | 7(2.66%) | 0(0.00%) |  |  |
| **#2** | 0(0.00%) | 4(1.52%) |  |  | 0(0.00%) | 0(0.00%) |  |  |
| **#3** | 1(0.38%) | 2(0.76%) |  |  | 0(0.00%) | 0(0.00%) |  |  |
| **#4** | 8(3.04 %) | 14(5,32%) |  |  | 4(1.52%) | 1(0.38%) |  |  |
| **#5** | 5(1.90%) | 10(3.80%) |  |  | 32(12.17%) | 4(1.52%) |  |  |
| **#6** | 5(1.90%) | 1(0.38%) |  |  | 4(1.52%) | 1(0.38%) |  |  |
| **#7** | 12(4.56%) | 17(6.46%) |  |  | 3(1.14%) | 22(0.76%) |  |  |
| **#8** | 0(0.00%) | 0(0.00%) |  |  | 0(0.00%) | 1(0.38%) |  |  |
| **#9** | 1(0.38%) | 5(1.90%) |  |  | 1(0.38%) | 1(0.38%) |  |  |
| **#10** | 21(7.98%) | 10(3.80%) |  |  | 6(2.28 %) | 31(11.79%) |  |  |
| **#11** | 19(7.22%) | 30(11.41%) |  |  | 9(3.42 %) | 16(6.08%) |  |  |
| **#12** | 5(1.90%) | 22(0.76%) |  |  | 1(0.38%) | 1(0.38%) |  |  |
| **#13** | 22(0.76%) | 1(0.38%) |  |  | 0(0.00%) | 0(0.00%) |  |  |
| **#14** | 0(0.00%) | 0(0.00%) |  |  | 0(0.00%) | 0(0.00%) |  |  |

Note:

(1) Lymph nodes and tumors were located on the same side of the trachea or esophagus;

(2) P^##^ value indicates the significance of differences between the characteristics in training and testing cohort;

(3) P-value indicates the significance of differences between the LN (+) group and LN (-) group.
